# Supplementary material for: In Vitro Comparison of the Internal Ribosomal Entry Site Activity from Rodent Hepacivirus and Pegivirus and Construction of Pseudoparticles
Source: Adv Virol. 2021 Jul 30;2021:5569844. doi: 10.1155/2021/5569844 (PMC8376455; doi:10.1155/2021/5569844)
Supplement: Supplementary Materials — (1) Illustration of plasmid construction as outlined in methods. (2) Supplementary analyses of level of RNA transcripts produced by viral 5′ UTRs. (3) MAVS cleavage and dsRNA analysis of full-length replicons. [file 5569844.f1.docx]

# Supplementary information

# In vitro comparison of the internal ribosomal entry site activity from rodent hepacivirus and pegivirus and construction of pseudoparticles

Stuart Sims^1^, Kevin Michaelsen^1^, Sara Burkhard^2^, Cornel Fraefel^1^

1. Institute of Virology, University of Zurich, Zurich, Switzerland
2. Department of Infectious Diseases, University Hospital of Zurich, Zurich, Switzerland

**Fig S1: Illustration of plasmid construction as outlined in methods.**

**Fig S2. Level of RNA transcripts produced by viral 5’ UTRs**

Hepa1-6 cells were transfected with monocistronic vectors containing either HCV, RHV, RHV1, RHV2, RHV3, RHV-rn1, RPgV, virus 5’UTR or the control plasmids, the first control contains the RHV1 virus 5’UTR but no upstream Pol I promoter and the second control plasmid contains a scrambled RHV1 5’UTR.

Cells were harvested at 72 h.p.t., RNA subtracted, and qRT-PCR performed, measuring the levels of mCitrine transcripts. Bar graphs show fold change in the levels of mCitrine transcripts to control plasmid, the HCV monocistronic vector (n≥10, mean±SEM of at least three independent experiments).

**Fig S3. MAVS cleavage and replication by full-length recombinant virus**

Huh7.5-MAVS-RFP cells transfected with full length RHV1 expression plasmid. Translocation of RFP to the nucleus is indicated by arrows (E).

BHK-21 cells transfected with full length recombinant RHV1 or RPGV expression plasmid containing mScarlet (Red) as in schematic, combined with dsRNA staining (Green) and DAPI (Blue) (F).
